# Progesterone elevation on the day of hCG trigger has detrimental effect on live birth rate in low and intermediate ovarian responders, but not in high responders

**DOI:** 10.1038/s41598-019-41499-1

**Published:** 2019-03-26

**Authors:** Ze Wu, Yunhua Dong, Yanping Ma, Yonggang Li, Lei Li, Na Lin, Yunxiu Li, Li Zhuan, Yun Bai, Xi Luo, Xiaomin Kang

**Affiliations:** 1 Department of Reproductive Medicine, The First People’s Hospital of Yunnan Province, Kunming, China; 2 Reproductive Medical Center of Yunnan Province, The Affiliated Hospital of Kunming University of Science and Technology, Kunming, China

## Abstract

Progesterone elevation (PE) on the day of hCG trigger is associated with decreased pregnancy outcome in fresh cycles. Evidence for this comes from overall patient estimates that mostly ignore different ovarian responses. To compare the impacts of PE on the day of hCG trigger on live birth rates (LBs) in low, intermediate and high ovarian responders and to explore the cut-off value for PE in different populations according to the ovarian response, we retrospectively analyzed a total of 2,351 patients receiving fresh assisted reproduction technology (ART) transfer cycles with GnRH agonist using a long or short protocol. Trend and multivariate logistic regression analyses were performed to identify the cutoff values of PE and to evaluate the effects of PE on LB rates (LBRs) in different ovarian responders. The study found that PE has a detrimental effect on LBRs in low to intermediate ovarian responders rather than in high responders. The cut-off values for PE were 1.0 ng/mL and 2.0 ng/mL for low and intermediate ovarian responders, respectively. The different associations between PE and LBRs according to ovarian response could more accurately predict the prognosis of the IVF cycle and could be used to optimize the treatment of patients undergoing *In Vitro* Fertilization (IVF)/ Intracytoplasmic Sperm Injection (ICSI).

## Introduction

Despite routine suppression of endogenous gonadotropins by gonadotropin-releasing hormone (GnRH) agonists, serum progesterone elevation (PE) on the day of human chorionic gonadotropin (hCG) trigger has been reported in controlled ovarian stimulation (COS) cycles in not only short protocols but also long protocols. The occurrence rates of PE have been reported to be 13% to 46% of *in vitro* fertilization/intracytoplasmic sperm injection (IVF/ICSI) cycles with GnRH agonists^1,2^. However, the possible effects of these subtle progesterone increases on pregnancy outcomes are controversial. Most studies have advocated that PE on the day of hCG trigger adversely affects pregnancy outcome^3–5^ due to its detrimental effect on the endometrium^6,7^ or the compromised quality of the oocyte^8,9^. A recent publication showed that the live birth rates (LBRs) were significantly lower in patients with both low (≤0.05 ng/mL) and high (≥1.5 ng/mL) progesterone levels on the day of hCG trigger^10^. However, the unfavorable effect of PE has not been found in other studies^11–13^. For most studies, the role of PE on the day of hCG trigger on pregnancy rates has been estimated through simple bivariate analyses, which are unable to control for confounders such as the number of oocytes, female age, or body mass index (BMI), and the available studies may actually underestimate the true effect of PE on pregnancy rates^14^. Furthermore, these varying results may also be attributed to the use of different arbitrary cut-off levels.

However, the effect of ovarian response on the association of PE with the probability of pregnancy outcomes remains unclear. Recent studies have shown that serum progesterone level is positively associated with ovarian response^3,15^ and oocyte number has been demonstrated to be correlated with PE^16^. Some studies have found that PE on the day of hCG trigger negatively influences pregnancy outcomes regardless of different ovarian responses, although in high responders at higher progesterone levels^3,15^. However, a more recent study reported that PE on the day of hCG trigger decreases the likelihood of pregnancy in low to normal responders, rather than in high responders^13^. It has been demonstrated that PE is associated with a higher number of oocytes^15,17^; more oocytes might indicate more available embryos, whereas fewer oocytes might indicate embryos that already have diminished implantation potential^6^. Thus, we believe that different ovarian responses might play a modulating role on the effect of PE on pregnancy outcomes. It is reasonable and important to assess the relationship between serum progesterone level and IVF/ICSI outcome according to different ovarian responses.

Hence, the aim of this study was to investigate the relationship between serum progesterone level on the day of hCG trigger and LBRs in patients with different ovarian responses. Moreover, the secondary aims were to identify the thresholds at which PE has a detrimental effect on LBRs in different ovarian responders and to explore the possible factors related to PE.

## Results

### Patient characteristics

The characteristics of the sample analyzed in this study are presented in Table 1. In the 2,351 patients undergoing their first IVF/ICSI cycles, there were 358 low ovarian responders, 1,649 intermediate ovarian responders, and 344 high responders. The basal FSH levels in these three groups were 7.28 ± 1.70, 6.99 ± 1.73, and 6.57 ± 1.73 IU/L, respectively. Several causes of infertility were present, including tubal factor endometriosis, PCOS, male factor, unexplained infertility, and mixed factors.

**Table 1:** Basic characteristics of the 2,351 patients undergoing their first IVF/ICSI cycle.

Variable	Low responders (n = 358)	Intermediate responders (n = 1649)	High responders (n = 344)
Age (years)	31.16 ± 3.67	30.51 ± 3.67	29.91 ± 3.57
BMI (kg/m^2^)	22.37 ± 3.29	21.71 ± 2.86	21.14 ± 2.61
Duration of infertility (y)	4.90 ± 3.29	4.79 ± 3.16	4.66 ± 2.79
Basal FSH (IU/L)	7.28 ± 1.70	6.99 ± 1.73	6.57 ± 1.73
**Cause of infertility (%)**			
Tubal factor	60.33	60.46	58.72
Endometriosis	1.67	1.45	0.87
PCOS	4.46	3.94	4.06
Male factor	17.31	19.10	20.05
Unexplained infertility	7.26	5.94	4.65
Mixed factors (%)	8.93	9.09	11.62
**ART (%)**			
IVF	83.79	79.50	81.39
ICSI	16.21	20.50	18.61
**Protocols (%)**			
Short protocol	86.03	81.14	80.23
Long protocol	13.97	18.86	19.77

BMI, body mass index; FSH, follicle-stimulating hormone; IVF, *in vitro* fertilization; ICSI, intracytoplasmic sperm injection. Values are means ± standard deviations, *n* (%) or *n*/total (n).

Table 2 presents the outcome of COS, fertilization, and ET. The gonadotropin dose was significantly different between the groups and was the highest in low ovarian responders (1929.52 ± 746.47 IU) and the lowest in high ovarian responders (1682.63 ± 669.46 IU) (P < 0.01). Significantly higher levels of serum progesterone E_2_ on the day of hCG trigger and more oocytes were retrieved in high ovarian responders. Compared with low ovarian responders, more oocytes were fertilized, and more good-quality embryos were implanted in intermediate and high ovarian responders. However, the rate of good-quality embryos did not reach statistical significance between the groups. The clinical pregnancy rates, implantation rates, and LBRs were 22.35%, 15.24%, and 16.48%, respectively, in low ovarian responders; 32.32%, 18.83%, and 24.15%, respectively, in intermediate ovarian responders; and 27.91%, 15.56%, and 20.05%, respectively, in high ovarian responders. A significant difference existed between low responders and intermediate ovarian responders (chi-square test *p* < 0.05 for each comparison).

**Table 2:** Outcomes of controlled ovarian stimulation, fertilization, and embryo transfer.

Variable	Low responders (n = 358)	Intermediate responders (n = 1,649)	High responders (n = 344)	*p* value
Duration of stimulation (d)	8.89 ± 1.92	9.05 ± 1.95	9.20 ± 1.77	<0.01^c^
Total dose of Gn (IU)	1929.52 ± 746.47	1795.21 ± 678.77	1682.63 ± 669.46	<0.01^a,b,c^
Endometrial thickness on hCG day(mm)	11.08 ± 2.1	11.33 ± 2.1	11.35 ± 2.0	NS
LH level on hCG day (IU/L)	4.73 ± 3.98	4.10 ± 3.32	3.53 ± 2.83	<0.05^a,b,c^
E_2_ level on hCG day (pg/mL)	2168.01 ± 1561.77	3873.55 ± 2118.79	6095.13 ± 2587.22	<0.01^a,b,c^
P level on hCG day (ng/mL)	1.03 ± 1.48	1.17 ± 1.10	1.47 ± 1.26	<0.01^a,b,c^
No. of oocytes retrieved	3.77 ± 1.20	11.40 ± 3.57	22.90 ± 4.23	<0.01^a,b,c^
No. of 2PN oocytes	2.53 ± 1.17	6.94 ± 3.38	14.27 ± 4.87	<0.01^a,b,c^
2PN rate (%)	907/1009 (89.89)	11440/13160 (86.93)	4909/5771 (85.06)	<0.01^a,b,c^
No. of good-quality embryos	1.31 ± 1.09	3.54 ± 2.58	7.12 ± 4.03	<0.01^a,b,c^
Good-quality embryos rate (%)	468/795 (58.87)	5836/9701 (60.16)	2450/4137 (59.22)	NS
No. of embryos transferred	1.72 ± 0.54	2.05 ± 0.38	2.06 ± 0.28	<0.01^a,c^
At least one good-quality embryo transferred rate (%)	266/358 (74.30)	1512/1649 (91.69)	331/344 (96.22)	<0.01^a,b,c^
No. of embryos frozen	1.65 ± 0.61	3.92 ± 2.35	7.56 ± 4.4	<0.01^a,b,c^
Clinical pregnancy rate (%)	80/358 (22.35)	533/1649 (32.32)	96/344 (27.91)	<0.01^a^
Implantation rate (%)	94/617 (15.24)	636/3378 (18.83)	110/707 (15.56)	<0.05^a,b^
Live birth rate (%)	59/358 (16.48)	398/1649 (24.15)	69/344 (20.05)	<0.01^a^

E_2_, estradiol; Gn, gonadotropin; hCG, human chorionic gonadotropin; LH, luteinizing hormone; NS, not significant.

Values are presented as the means ± standard deviations or counts (%). ANOVA or chi-square test.

^a^Significant difference between low and intermediate ovarian responders.

^b^Significant difference between intermediate and high ovarian responders.

^c^Significant difference between low and high ovarian responders.

### Relationships between serum progesterone levels and LBRs

**Figure 1:**
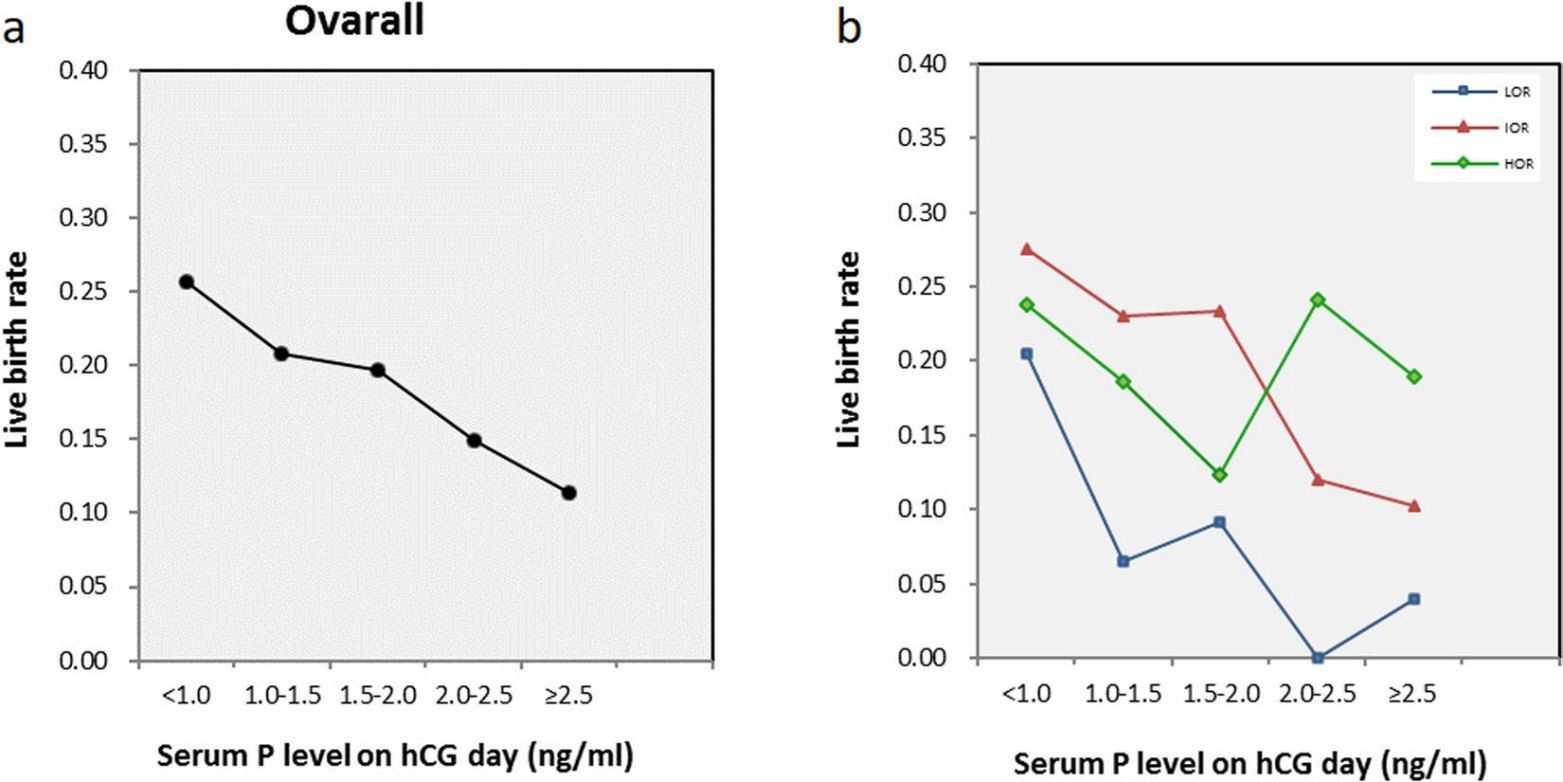
Relationships between serum progesterone levels and live birth rates. The relationships between serum progesterone levels and live birth rates in (**a**) all patients and in (**b**) low ovarian responders (LOR), intermediate ovarian responders (IOR), and high ovarian responders (HOR).

Figure 1 depicts the relationships between serum progesterone levels and LBRs in fresh IVF/ICSI cycles. Figure 1a shows that the LBRs in all patients decreased when the progesterone level was ≥1.0 ng/mL, and the same trend was observed in low ovarian responders (Fig. 1b); in intermediate ovarian responders, the decreased LBRs appeared only under the condition of progesterone level ≥2.0 ng/mL (Fig. 1b). However, the analysis failed to identify a detrimental progesterone cut-off value in high ovarian responders (Fig. 1b).

The OR (95% confidence interval [CI]) for LBR for each group compared with the lowest progesterone group is listed in Table 3, showing the difference of relative change in OR between intervals, confirming the nonlinear relationship between LBRs and intervals of serum progesterone level. Furthermore, a statistically significant difference was observed when the 1.0–1.5 ng/mL group was compared with the <1.0 ng/mL group (25.7% vs 20.8%, *P* = 0.028) in the overall study group. In the low ovarian response group, the difference was statistically significant between the 1.0–1.5 ng/mL and the <1.0 ng/mL intervals *(P* = 0.024); in intermediate ovarian responders, the differences were statistically significant between the 2.0–2.5 ng/mL and <1.0 ng/ml intervals (*P* = 0.004) and the 2.0–2.5 ng/mL and the 1.5–2.0 ng/mL intervals (*P* = 0.047), respectively. In high ovarian responders, although the LBRs decreased numerically with progesterone ≥1.5 ng/mL, no statistically significant differences were observed between the 1.50–1.99 ng/mL and <1.00 ng/mL intervals (OR: 0.447; *P* = 0.07) and the 1.5–2.0 ng/mL and 1.0–1.5 ng/mL intervals (OR: 0.839; *P* = 0.314). These data suggest that a serum progesterone level of 1.0 ng/mL or 2.0 ng/mL may represent the cut-off value to define PE where there was a detrimental effect of progesterone on LBRs in patients with low or intermediate ovarian response, respectively, but no detrimental progesterone cut-off value was identified in patients with a high ovarian response.

**Table 3:** Live birth rates according to serum progesterone levels.

P level (ng/mL)	Overall OR (95% CI) p value	Low ovarian responders OR (95% CI) p value	Intermediate ovarian responders OR (95% CI) p value	High ovarian responders OR (95% CI) p value
<1.0	—	—	—	—
1.0–1.5	0.761 (0.596–0.971) 0.028	0.271 (0.081–0.908) 0.024	0.787 (0.597–1.037) 0.089	0.730 (0.371–1.436) 0.371
1.5–2.0	0.708 (0.507–0.989) 0.042	0.389 (0.088–1.715) 0.197	0.807 (0.552–1.179) 0.267	0.447 (0.184–1.076) 0.071
2.0–2.5	0.507 (0.298–0.861) 0.011	0 0.603	0.382 (0.194–0.754) 0.004	1.016 (0.396–2.605) 0.973
≥2.5	0.373 (0.230–0.604) 0.001	0.162 (0.021–1.225) 0.058	0.299 (0.158–0.567) 0.000	0.315 (0.184–1.793) 0.518

CI, confidence interval; OR, odds ratio. Data are expressed as ORs (95% CIs) for each of the serum progesterone levels compared with the lowest P group (<1.0 ng/mL).

Considering potential factors that might influence LBR, multivariate logistic regression analysis in different ovarian responders was conducted separately. The results confirmed that the progesterone level had a negative effect on LBR in low and intermediate ovarian responders, but not in high ovarian responders (Table 4).

**Table 4:** Factors associated with live birth rate in different ovarian response groups by logistic regression.

Variable	Low responders COR (95% CI) AOR (95% CI)	Intermediate responders COR (95% CI) AOR (95% CI)	High responders COR (95% CI) AOR (95% CI)
Age (y)	0.938 (0.870–1.010)	0.996 (0.966–1.027)	0.957 (0.889–1.030)
BMI (kg/m^2^)	1.020 (0.940–1.110)	1.047 (1.008–1.089)^*^	0.973 (0.879–1.078)
Duration of stimulation(d)	0.964 (0.880–1.055)	0.980 (0.945–1.016)	0.916 (0.827–1.014)
Basal FSH (mIU/mL)	0.982 (0.834–1.155)	0.956 (0.897–1.020)	1.011 (0.868–1.179)
Duration of stimulation(d)	1.037 (0.900–1.194)	1.007 (0.952–1.066)	0.965 (0.827–1.125)
Total dose of Gn (IU)	1.000 (1.000–1.000)	1.000 (1.000–1.000)	1.000 (1.000–1.000)
LH level on hCG day (mIU/mL)	0.967 (0.897–1.041)	0.986 (0.953–1.021)	1.021 (0.933–1.18)
E_2_ level on hCG day (pg/mL)	1.000 (1.000–1.000)	1.000 (1.000–1.000)	1.000 (1.000–1.000)
No. of oocyte retrieved	1.130 (0.887–1.438)	0.995 (0.964–1.027)	1.052 (0.994–1.115)
2PN rate (%)	7.730 (1.336–43.724)^*^	3.522 (1.914–6.481)^**^	7.007 (1.007–45.586)^*^
No. of good-quality embryos	1.611 (1.257–2.065)^**^	1.107 (1.061–1.155)^**^ 1.081 (1.032–1.132)^**^	1.072 (1.004–1.144)^*^
At least one good-quality embryo transferred rate (%)	3.567 (1.478–8.604)^**^	1.950 (1.197–3.178)^**^	3.103 (0.396–24.279)
Endometrial thickness (mm) on hCG day	1.167 (1.025–1.329)^*^	1.116 (1.055–1.179)^**^ 1.104 (1.043–1.168)^**^	1.064 (0.937–1.209)
Progesterone level on hCG day (ng/mL)	0.570 (0.344–0.942)^*^ 0.538 (0.306–0.946)^*^	0.671 (0.569–0.793)^**^ 0.686 (0.580–0.810)^**^	0.861 (0.653–1.135)

AOR, adjusted odds ratio; BMI, body mass index; CI, confidence interval; COR, crude odds ratio; E_2_, oestradiol; FSH, follicle-stimulating hormone; Gn, gonadotropin; hCG, human chorionic gonadotropin.

^*^P < 0.05,

^**^P < 0.01.

### Multivariate analysis of factors involved in PE

After all of the variables were included in the multivariate regression analysis, we identified ten possible factors related to PE that indicated that in all three ovarian responders, the E_2_ and LH levels on the day of hCG trigger were all associated with PE (Table 5).

**Table 5:** Multivariate regression analysis of factors related to progesterone elevation.

Variable	Low ovarian responders OR (95% CI) *p* value	Intermediate ovarian responders OR (95% CI) *p* value	High ovarian responders OR (95% CI) *p* value
Age (y)	0.943 (0.875–1.017) 0.129	0.985 (0.943–1.030) 0.518	0.986 (0.893–1.089) 0.783
BMI (kg/m^2^)	0.924 (0.845–1.010) 0.082	0.887 (0.833–0.945) <0.001	1.019 (0.883–1.175) 0.801
Basal FSH (mIU/mL)	0.912 (0.778–1.070) 0.259	0.961 (0.873–1.059) 0.425	1.299 (1.009–1.175) 0.042
Basal E_2_ (pg/mL)	1.011 (1.000–1.023) 0.054	1.003 (0.995–1.011) 0.439	1.006 (0.988–1.025) 0.514
Long Protocol versus Short Protocol	1.607 (0.499–5.180) 0.427	0.765 (0.404–1.449) 0.411	0.349 (0.059–2.066) 0.246
Duration of stimulation(d)	1.056 (0.893–1.249) 0.524	1.117 (1.033–1.208) 0.006	1.201 (0.959–1.505) 0.111
Total dose of Gn (IU)	1.000 (1.000–1.001) 0.039	1.000 (1.000–1.000) 0.143	1.000 (0.999–1.001) 0.971
LH level on hCG day (mIU/mL)	1.186 (1.104–1.274) <0.001	1.151 (1.100–1.205) <0.001	1.208 (1.075–1.358) 0.002
E_2_ level on hCG day (pg/mL)	1.000 (1.000–1.000) 0.038	1.000 (1.000–1.000) 0.013	1.000 (1.000–1.000) 0.014
Oocytes retrieved	1.074 (0.855–1.350) 0.537	1.077 (1.029–1.127) 0.001	1.000 (0.917–1.089) 0.993

AOR, adjusted odds ratio; BMI, body mass index; CI, confidence interval; COR, crude odds ratio; Gn, gonadotropin.

### Results of comparisons between subgroups

Demographic and clinical characteristics were compared among the subgroups within the low ovarian responders (Table 6) and the intermediate ovarian responders (Table 7).

**Table 6:** Comparisons of clinical characteristics and cycle outcomes in low ovarian responders with and without PE (≥1.0 ng/mL).

Variable	Non-PE (<1.0 ng/mL)	PE (≥1.0 ng/mL)	*P* value
Age (y)	31.20 ± 3.77	31.05 ± 3.41	NS
BMI (kg/m^2^)	22.73 ± 3.37	21.42 ± 2.89	0.001
Duration of infertility (y)	4.79 ± 3.19	5.18 ± 3.23	NS
Basal FSH (mIU/mL)	7.28 ± 1.70	7.29 ± 1.72	NS
Duration of stimulation(d)	8.94 ± 1.97	8.74 ± 1.77	NS
Total dose of Gn (IU)	1901.64 ± 681.80	2002.46 ± 893.60	NS
Endometrial thickness (mm) on hCG day	11.10 ± 2.07	11.03 ± 2.10	NS
LH on hCG day (pg/mL)	4.01 ± 3.58	6.62 ± 4.34	<0.001
E_2_ level on hCG day (pg/mL)	1959.01 ± 1544.46	2714.86 ± 1479.21	<0.001
No. of oocytes retrieved	3.72 ± 1.23	3.91 ± 1.14	NS
No. of 2PN oocytes	2.54 ± 1.16	2.52 ± 1.20	NS
2PN rate (%)	658/727 (90.51)	249/282 (88.29)	NS
Good-quality embryos	1.34 ± 1.08	1.21 ± 1.14	NS
Good-quality embryos rate (%)	348/614 (56.67)	120/234 (51.28)	NS
At least one good-quality embryo transferred rate (%)	199/259 (76.83)	67/99 (67.68)	NS
No. of embryos transferred	1.73 ± 0.54	1.70 ± 0.56	NS
Clinical pregnancy rate (%)	70/259 (27.03)	10/99 (10.10)	0.01
Implantation rate (%)	85/449 (18.93)	9/168 (5.36)	0.001
LBR(%)	53/259 (20.46)	6/99 (6.06)	0.001

BMI, body mass index; E2, oestradiol; FSH, follicle-stimulating hormone; Gn, gonadotropin; hCG, human chorionic gonadotropin; LBR, live birth rate; NS, not significant. Values are presented as the means ± standard deviations or counts (%). Kruskal-Wallis test or chi-square test. NS = not significant.

**Table 7:** Comparisons of clinical characteristics and cycle outcomes in intermediate ovarian responders with and without progesterone elevation (≥2.0 ng/mL).

Variable	Non-PE (<2.0 ng/mL)	PE (≥2.0 ng/mL)	*P* value
Age (y)	30.54 ± 3.68	30.32 ± 3.65	NS
BMI (kg/m^2^)	21.82 ± 2.88	20.89 ± 2.58	<0.001
Duration of infertility (y)	4.83 ± 3.17	4.50 ± 3.08	NS
Basal FSH (mIU/mL)	6.98 ± 1.74	7.06 ± 1.69	NS
Duration of stimulation(d)	9.03 ± 2.01	9.20 ± 1.45	NS
Total dose of Gn (IU)	1792.94 ± 675.63	1812.98 ± 704.46	NS
Endometrial thickness (mm) on hCG day	11.36 ± 2.02	11.06 ± 1.98	NS
LH on hCG day (pg/mL)	3.94 ± 3.25	5.38 ± 3.59	<0.001
E_2_ on hCG day (pg/mL)	3750.67 ± 2002.30	4834.29 ± 2690.31	<0.001
No. of retrieved oocytes	11.30 ± 3.56	12.30 ± 3.59	<0.001
No. of 2PN oocytes	6.92 ± 3.37	7.07 ± 3.47	NS
2PN rate (%)	10117/11599 (87.22)	1323/1561 (84.54)	0.007
Good-quality embryos	3.55 ± 2.59	3.42 ± 2.53	NS
Good-quality embryos rate (%)	5197/9476 (54.84)	639/1226 (52.12)	NS
At least one good-quality embryo transferred rate (%)	1343/1462 (91.86)	169/187 (90.34)	NS
No. of embryos transferred	2.05 ± 0.38	2.01 ± 0.39	NS
Clinical pregnancy rate (%)	500/1462 (34.19)	33/187 (17.64)	<0.001
Implantation rate (%)	603/3002 (20.09)	33/376 (8.78)	<0.001
LBR(%)	377/1462 (25.79)	21/187 (11.23)	<0.001

BMI, body mass index; E2, oestradiol; FSH, follicle-stimulating hormone; hCG, human chorionic gonadotropin; LBR, live birth rate; NS = not significant; PE, progesterone elevation. Values are presented as the means ± standard deviations or counts (%). Kruskal-Wallis test or chi-square test.

In the low ovarian responders, BMI was higher in the <1.0 ng/mL subgroup than in the ≥1.0 ng/mL subgroup. Female age, basal FSH level, duration of infertility, duration of COS, gonadotropin dosage, endometrial thickness on the day of hCG trigger, number of oocytes retrieved, 2PN oocytes, good-quality embryos/rate, at least one good-quality embryo transferred rate, and number of embryos transferred and frozen did not differ significantly between the two subgroups (Table 6). When serum progesterone was elevated, the serum LH, E2 levels, and the number of oocytes retrieved were increased; however, the clinical pregnancy rate (10.10% versus 27.03%, *p* = 0.01), implantation rate (5.36% versus 18.93%, *p* = 0.001), and LBR (6.06% versus 20.46%, *p* = 0.001) were significantly decreased.

In the intermediate ovarian responders (Table 7), patients without PE (<2.0 ng/mL) or with PE (≥2.0 ng/mL) were similar regarding female age, basal FSH levels, duration of stimulation, total dose of gonadotropin, endometrial thickness, 2PN oocytes, good-quality embryos/rate, at least one good-quality embryo transferred rate, and number of embryos transferred and frozen. The larger number of retrieved oocytes and higher serum LH and E_2_ levels observed in the ≥2.0 ng/mL subgroup may indicate that this subgroup had a better ovarian response. However, this subgroup had significantly lower clinical pregnancy rate (17.6 4% vs. 34.19%, *p* < 0.001), implantation rate (8.78% vs. 20.09%, *p* < 0.001), and LBR (11.2 3% vs 25.79%, *P* = 0.001) compared with the <2.0 ng/mL subgroup. In addition, BMI was significantly higher in the <2.0 ng/mL subgroup (21.82 ± 2.88 vs 20.89 ± 2.58, *p* < 0.001).

## Discussion

At present, for the high progesterone population, the common practice is to cancel the fresh ET and freeze-all to achieve a good pregnancy outcome in subsequent frozen ET (FET) cycles. This approach is simple, but is it appropriate for every patient? To test this question, we retrospectively analyzed the LBRs of PE for different populations according to ovarian responses.

The current studies showed that low LBRs are only associated with serum PE in low and intermediate ovarian responders rather than in high ovarian responders. The thresholds for serum PE were 1.0 ng/mL and 2.0 ng/mL, respectively, for low and intermediate ovarian responders. However, the analysis failed to identify a detrimental cut-off value in high ovarian responders. This finding strongly suggests that LBRs should be considered in association with the PE and ovarian response; it should be adopted a more accurate estimation of the real impact of PE on the LBRs in the IVF cycle is needed and could provide a reference for clinical management decisions for patients with PE.

The effect of PE on the hCG trigger day remains uncertain, although large amounts of observational data have been published^2,8–10,18^. Controversy remains due to the distinct definition of PE, the different statistical methods used, the different characteristics of patients recruited, and the retrospective nature of studies analyzing the relationship between PE and clinical outcomes^19^. More recently, Venetis *et al*.^14^ strongly suggested that a multivariable approach should be used to more accurately predict the real effect of PE on the LBR of patients after IVF treatment. Multivariate regression analysis aims to eliminate the influence of confounding factors or other forms of deviation from mathematics, to better approach the real influence of independent variables on dependent variables^20,21^. However, no matter which method is used, most scholars believe that the adverse effects of serum PE on the IVF/ICSI outcome are consistent^3^.

The pathogenesis of serum PE in GnRH agonist cycles is complicated. Despite the elimination of the LH surge in 95% to 98% of patients since the introduction of GnRH^22,23^, PE is still a frequent phenomenon in ovarian stimulation cycles. Most of the studies have been conducted in GnRH agonist long protocols or/and GnRH antagonist protocols^3,4,13,15,24,25^. The occurrence of PE and its effect on clinical outcomes in short protocols have been poorly understood, with few papers published before now^19,26^. To the best of our knowledge, the current study included the largest number of patients (n = 1922) treated with a short protocol, strengthening the reliability of our conclusion. The results of our multivariate analysis showed that LH and E_2_ levels on the day of hCG trigger were correlated with PE in all ovarian response groups. The LH and E_2_ levels on the day of hCG trigger were markedly higher in the PE group than in the non-PE group. In contrast, a recent study by Koo *et al*.^27^ showed that the LH level was markedly lower in the PE group than in the non-PE group. This discrepancy may result from different protocols. In our study, GnRH agonist protocols, mainly short protocols (81.75% of the total study population), were used, and PE might be the consequence of a relatively high LH level resulting from the flare-up effect of GnRH agonist, adding further evidence that LH might also be involved in the occurrence of PE^19^. The elevated P might be attributed to an excess number of follicles, each one producing a normal amount of progesterone for the late follicular phase. Thus, the excess of proliferating granulosa cells leads to an increase in progesterone production independent of LH exposure^28,29^, with each individual follicle contributing to the collective concentration observed in circulation^30^. Finally, higher serum E_2_ levels and numbers of oocytes retrieved were associated with elevated progesterone concentrations^15,29,31^. Therefore, it is reasonable to conclude that the level of progesterone at the end of COS depends on the ovarian response, and the patients with high ovarian responses tend to be higher^32^.

Despite the fact that the precise mechanism of PE on IVF pregnancy outcome is unclear, endometrial receptivity and embryo quality are two key factors to the success of implantation^33^. Most studies^2,15,19,28,34^ have shown that PE affects endometrial receptivity in fresh ET cycles. PE is likely to promote the endometrium without affecting the embryo, leading to the dyssynchrony between the embryo and the endometrium, which may lead to a decrease in implantation rate, thereby reducing the pregnancy rate^7^. Embryo cryopreservation could rescue cycles with PE^35^. Patients with PE could achieve a good pregnancy outcome in subsequent embryo transfer (FET) cycles, and the result was similar to those patients without PE, confirming this theory^2,15,34^.

There are some limitations and drawbacks in this study. First of all, although we have a large number of patients, and the sample sizes of the long and short protocols were asymmetrical. Due to the methodological restrictions of retrospective design, this asymmetry may lead to certain flaws or bias. Xu *et al*. showed that progesterone levels affect LBR regardless of ovarian response^15^. Conversely, our research suggests that progesterone does not affect LBR in high responders’ group. This may be due to the fact that we have a small sample size. Second, there are certain differences in the downregulation methods, types and dosages of gonadotropins, amounts of hCG for triggering, and the duration of COS. Especially for patients with PCOS, the FSH starting dose is different from others^36,37^. We cannot clearly know how much these differences will affect the outcome. Third, according to the recent evidences, the supplementation with oral inositol is able to improve oocyte quality and, in this way, also reproductive outcomes of IVF procedures^38,39^. Transvaginal ultrasound guidance of the transfer significantly increases the percentage of pregnancies^40,41^. However, since we have not made such these attempts, it is not clear whether there will be any influences in the reference population. Furthermore, although we adopted a multivariate analysis to estimate the relationships between PE and LBR according to different ovarian responses, the characteristics of this retrospective study may lead to a deviation in the interpretation of data^3^.

## Methods

### Study population and ovarian stimulation

The research project and the protocols were approved by the Medical Ethics Committee of The First People’s Hospital of Yunnan Province. The signed informed consent was obtained from all patients. All the data used in the study were collected from patients undergoing routine and standard IVF treatment in a licensed center without any additional intervention. All human study methods were performed in accordance with the relevant guidelines and regulations.

This is a noninterventional retrospective analysis of all IVF/ICSI cycles in women who started their first IVF/ICSI cycles with GnRH agonist treatment in the Reproductive Medicine Centre, the First People’s Hospital of Yunnan Province, from January 2008 to March 2011 (after March 2011, the fresh ET and freeze-all were cancelled for patients with progesterone level ≥2.0 ng/ml). The inclusion criteria included the following: women younger than 38 years, basal serum FSH on day 2 < 10 IU/L, day 3 embryo transfer. Only the first stimulation cycle for each woman was considered. No fresh ET was cancelled in the case of increasing progesterone levels. COS was performed in patients using either a short protocol or a long protocol, and other options were ignored.

A total of 2351 IVF/ICSI-ET cycles were performed. Patients underwent COS using either a GnRH agonist short protocol (n = 1,922) or a long protocol (n = 429); the details have been published previously^2^. Briefly, GnRH analogues short protocol was administered from day 2 of menstruation (0.1 mg Diphereline; Beaufour Ipsen, France). Ovarian stimulation started on day 3 with 150–300 IU recombinant FSH/HMG (75 IU Gonal-F; Serono, Switzerland; human menopausal gonadotrophin (HMG; 75 IU; Livzon Pharmaceutical, China). On day 2, prior to administering GnRH analogues, the serum FSH, estradiol and progesterone concentrations were measured, and the recombinant FSH/HMG dose was adjusted according to the ovarian response. Patients were evaluated by ultrasonography and serum estradiol, LH and progesterone concentrations from day 8 until the day of the HCG trigger. Specialized ultrasound technicians monitored the ovarian response by vaginal ultrasonography. HCG (5000 IU per ampoule, Profasi; Serono) 5000–10,000 IU was given i.m. when the two leading follicles reached a mean diameter of 18 mm. Transvaginal ultrasound-guided oocyte retrieval was scheduled 34–36 h after HCG trigger.

For the GnRH analogues long protocol, ovarian stimulations were performed with recombinant FSH/HMG after pituitary downregulation with GnRH analogues. The pituitary suppression was achieved with either daily 0.1 mg triptorelin (Diphereline) from the mid-luteal phase of the cycle preceding the treatment cycle, and after ovarian suppression was achieved, the dose of triptorelin was reduced to 0.05 mg until the day of HCG trigger; or a 1.25–1.875 mg dose of triptorelin (3.75 mg per ampoule Diphereline) was administered on day 21 of the cycle once.

Because this was a retrospective study, no specific criteria for the selection of the stimulation protocol were defined; the choice of COS protocol was made by the treating physician and was largely dependent on the method at the time. Before 2010, the short protocol was prevalent; later, the long protocol was gradually widely used in our center’s IVF/COS practice.

Fertilization of the aspirated oocyte was performed *in vitro* by either conventional IVF or ICSI depending on the semen parameters. Embryo assessment was performed for the number and regularity of blastomeres and the degree of embryonic fragmentation on the cleavage stage; the criteria were described previously^42^. Grade I and grade II embryos were good-quality embryos.

Fresh embryo transfers were performed on day 3 after oocyte retrieval. The additional good-quality embryos were cryopreserved for subsequent FET cycles. The number of embryos transferred ranged from one to three according to national regulations. Transferring three embryos was considered only in women older than 35 years. The luteal phase was supported with natural progesterone in oil (Progesterone Injection; XianJu Pharma, China), 60 mg progesterone intramuscularly daily from the day of oocyte retrieval until the 10th week of gestation, if pregnancy occurred.

### Outcome variables

The primary outcome variable for this study was LBR, and the secondary outcome variables were clinical pregnancy rate and implantation rate. Live birth was defined as the delivery of a viable infant after 28 weeks of gestation. Clinical pregnancy was defined as the presence of one or more gestational sacs detected by an ultrasound scan performed 5 weeks after embryo transfer. Implantation rate was defined as the number of gestational sacs (both within and outside the uterine cavity) seen on the ultrasound divided by the total number of embryos transferred.

### Hormone measurements

Serum progesterone, LH, and E_2_ levels were measured routinely prior to initiation (on day 2 to 3 of the cycle), during ovarian stimulation, and on the morning of the day of hCG trigger. Samples were tested using a Beckman-Coulter Unicel DxI 800 Access Analyzer based on chemiluminescence and commercially available kits (Beckman-Coulter, USA). Analytical sensitivity was 0.1 ng/mL for progesterone, 0.2 mIU/mL for LH, and 20 pg/mL for E_2_. Intra- and interassay precision rates at the concentrations, expressed as coefficients of variance, were 9.57% and 11.19% for progesterone, 3.8% and 6.4% for LH, and 12.0% and 21.0% for E_2_, respectively.

### Statistical analysis

Patients were categorized into three groups based on the number of oocytes retrieved: low ovarian responder (<6 oocytes obtained), intermediate ovarian responder (6 to 18 oocytes obtained), and high ovarian responder (>18 oocytes obtained)^13^. To avoid bias of the results by assuming that any relationship between serum progesterone levels and LBRs may be linear, in all three ovarian response groups, patients were then divided into five distinct groups according to their serum progesterone levels on the day of hCG trigger: <1.0, 1.0–1.5, 1.5–2.0, 2.0–2.5, and ≥2.5 ng/mL. The cut-off levels were chosen to provide equal intervals focused around the threshold values used across previous studies^3,9,43,44^.

LBRs were calculated for each progesterone interval. To identify the cut-off value of PE, trend analysis was performed using the Xu *et al*.^15^ method. Briefly, the OR and 95% confidence interval (CI) of LBRs for each progesterone interval, compared with the lowest progesterone group, were calculated. Factors related to PE were assessed using a multivariate analysis.

The distribution of continuous variables is expressed as the mean and standard deviation (SD). Categorical variables are presented as proportions and percentages of the total. Student’s *t*-test, one-way analysis of variance (ANOVA) or the Kruskal-Wallis test for continuous data and the chi-square test for categorical variables were used for data analysis. The statistical analysis was performed using the Statistical Program for Social Sciences version 19.0 (SPSS, Chicago, IL, USA). A value of *P* < 0.05 was considered to be statistically significant.

## Conclusions

Our analysis shows that PE has detrimental effects on LBRs in low to intermediate ovarian responders but did not compromise LBRs in high responders. The different associations between PE and LBRs according to ovarian response could more accurately forecast the prognosis of the *in vitro* fertilization cycle and could be used to optimize the treatment of IVF/ICSI patients. For patients with low ovarian responses, the critical value of PE should be set as 1.0 ng/mL, while for intermediate ovarian responders, the critical value is 2.0 ng/mL. It is necessary to combine the ovarian response and PE value to decide whether to cancel the fresh ET. Regarding high responders, the risk of OHSS should be paid more attention rather than PE on the day of hCG trigger.

## Data Availability

The data that support the findings of this study are available from the First People’s Hospital of Yunnan Province, but restrictions apply to the availability of these data, which were used under license for the current study, and so are not publicly available. Data are however available from the authors upon reasonable request and with permission of the First People’s Hospital of Yunnan Province.
